# The potential role of the gingival crevicular fluid biomarkers in the prediction of pregnancy complications

**DOI:** 10.3389/fmed.2023.1168625

**Published:** 2023-06-05

**Authors:** Maryam Abouzaid, Nourhan Howidi, Zahi Badran, Ghada Mohammed, Noha A. Mousa

**Affiliations:** ^1^College of Medicine, University of Sharjah, Sharjah, United Arab Emirates; ^2^Periodontology Unit, College of Dental Medicine, University of Sharjah, Sharjah, United Arab Emirates; ^3^Department of Clinical Sciences, College of Medicine, University of Sharjah, Sharjah, United Arab Emirates

**Keywords:** gingival crevicular fluid, biomarkers, adverse pregnancy outcomes, pregnancy complications, detection, periodontal disease

## Abstract

Early and minimally invasive methods are required to predict the risk of multiple adverse pregnancy outcomes. A potential technique with growing interest utilizes the gingival crevicular fluid (GCF), a physiological serum exudate found in the healthy gingival sulcus and in the periodontal pocket in inflammatory conditions. Analysis of biomarkers in the GCF is a minimally invasive method that can be feasible and cost-effective. The potential use of GCF biomarkers along with other clinical indicators in early pregnancy may provide reliable predictors of several adverse pregnancy outcomes, therefore, reducing both maternal and fetal morbidities. Various studies have reported that increased or decreased concentrations of different biomarkers in GCF are associated with a high risk of developing pregnancy complications. In particular, such associations have been commonly demonstrated with gestational diabetes, pre-eclampsia, and pre-term birth. However, limited evidence is available regarding other pregnancy complications such as preterm premature rupture of membranes, recurrent miscarriage, small for gestational age, and hyperemesis gravidarum. In this review, we discuss the reported association between individual GCF biomarkers and common pregnancy complications. Future research is required to provide more solid evidence of the predictive value of those biomarkers in estimating women’s risk for each disorder.

## Introduction

Despite the continuous improvements in the accessibility of women to pregnancy care, the unpredictability of many serious pregnancy complications remains challenging (1–3). Most of the obstetric complications follow an acute or sudden course, and in developing clinical settings are often diagnosed when the maternal or fetal condition has already been compromised (4–6). Therefore, considerable effort has been ongoing to identify reliable biomarkers for the early diagnosis or prediction of critical adverse pregnancy outcomes. The availability of such biomarkers is necessary for decision-making by obstetricians and to aid the development of appropriate preventive interventions. In the current clinical practice, only a few tests are widely used to detect the risk for pregnancy complications. Among those routine tests, the oral glucose tolerance test (OGTT) is used between 24 and 28 weeks of gestation for the diagnosis of gestational diabetes mellitus (GDM), and the vaginal fluid fetal fibronectin is used for the diagnosis of preterm birth before 34 weeks of gestation in symptomatic women (7, 8). As such, both tests are performed late in pregnancy, require blood samples or vaginal examination, and have more of a diagnostic rather than a predictive value. Accordingly, the availability of minimally invasive assays for biomarker identification in risk assessment would enhance their acceptability among pregnant women, leading to broader adoption of these assays for early screening purposes.

Urinary biomarkers have been traditionally used for diverse screening and diagnostic purposes. Urine dipstick tests are currently applied to detect a limited set of general biomarkers, such as proteins, glucose, and nitrites, which are mainly used to diagnose preeclampsia, gestational diabetes, and urinary tract infections, respectively (9–11). Other urine-based assays remain investigative; for instance, the urinary levels of 8-oxo-7,8-dihydro-2′-deoxyguanosine (8-oxodG) were proposed to detect pregnancy conditions associated with oxidative stress, such as the diagnosis of small for gestational age (12). Stool biomarker analysis was also used to investigate the relation of *H. pylori* antigen and gut microbiota dysbiosis with hyperemesis gravidarum, preeclampsia, and preterm birth (13–16). Although these non-invasive biomarker detection methods are currently under investigation, they have limitations such as low accuracy, low positive predictive value, low sensitivity, low specificity, and/or insufficient information for incorporation into clinical practice (9, 10, 15, 16).

Similarly, saliva-based biomarker analysis has gained interest in detecting markers such as glucose, visfatin, resistin, and uric acid (17–20). Salivary uric acid and placental growth factor (PlGF) have been investigated for their association with the risk of preeclampsia (21, 22). Likewise, salivary levels of progesterone and estriol have been investigated in regard to the risk of preterm birth (23, 24). In addition, salivary *H. Pylori* genome, cortisol, and dehydroepiandrosterone sulphate (DHEA-S) have been studied in relation to the risk of developing hyperemesis gravidarum (25, 26).

In addition to the above-mentioned conventional body fluids, the gingival crevicular fluid (GCF) has gained growing research interest. It is a physiological body fluid and inflammatory serum exudate, typically found in the healthy gingival sulcus and in the periodontal pocket in inflammatory states. It is composed of serum, inflammatory mediators, antibodies, tissue breakdown products, electrolytes, and bacteria from the subgingival plaque. Although GCF has been utilized for analyzing biochemical parameters to identify early periodontal disease, it has been recently presented as a minimally invasive source for numerous biomarkers that can be used to detect several systemic diseases (27).

This review aims to evaluate the current evidence regarding the association between GCF-based biomarkers and common pregnancy complications. We discuss the potential application of GCF biomarkers in risk prediction or early diagnosis of pregnancy-specific disorders.

## Gingival crevicular fluid: methods and features

**Figure 1:**
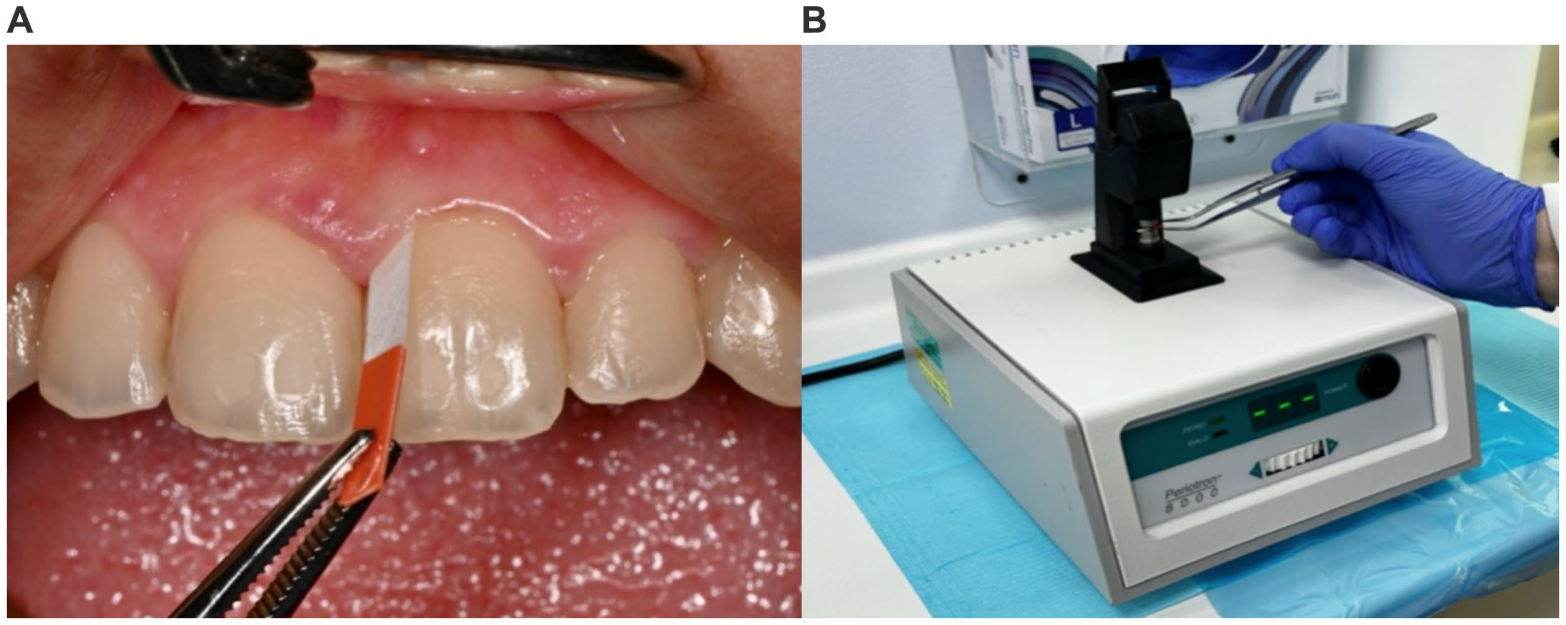
Gingival crevicular fluid sampling and use of the Periotron^®^ device. **(A)** The sample site is isolated with cotton rolls and air-dried, and a filter paper strip (Perio^®^ paper) is placed in the gingival sulcus for 30 s. **(B)** After removal of the strip, it is inserted into the Periotron 8000^®^ device (OraFlow Inc., NY, USA) to measure the volume of fluid collected.

The GCF can be obtained through several minimally invasive methods including, absorbent filter paper strips, pre-weighed twisted threads, micropipettes, or crevicular washings. Filter paper strips are the most common, efficient, easiest, and least traumatic method to obtain a GCF sample (28). The paper strip is inserted into the sulcus of the periodontal pocket and is left for 30 to 60 sixty seconds for the adsorption of GCF by the strip (29). Afterwards, the paper strip is placed in a specific device to measure the flow rate and volume of the collected GCF allowing the concentration of the biomarker to be calculated (Figure 1). Further analysis is done for biomarker detection using a biomarker-specific assay, such as the enzyme-linked immunoassay (ELISA) (30).

Practical feasibility is one of the most attractive features of the GCF, making it a unique minimally invasive, fast, patient-and practitioner-friendly source for biomarker assays (31, 32). Under physiological conditions, the gingival sulcus contains a minimal amount of GCF. However, during systemic or local inflammatory conditions, the fluid increases both in volume and flow rate and is converted from an inflammatory exudate to a transudate, in which biomarkers are present in substantially increased concentrations (27, 31–33). In such conditions, the GCF volume along with elevated biomarker levels serve as a general indicator of inflammation and subsequently an important tool for assessing health and disease conditions (27, 32, 34). Therefore, GCF can be of significant importance in proteomic studies, especially when linked to adverse pregnancy outcomes (31, 32).

Despite that GCF is considered one of the constituents of saliva, the latter contains additional components such as serum, nasal or bronchial secretions, enzymes, microbial and antimicrobial products, and epithelial, inflammatory, or food debris. Unlike salivary components which typically reflect the activity of all oral sites and the inflammatory condition of the mouth, GCF, on the other hand, can be considered periodontium-specific. Multiple biomarkers have been identified in GCF that were linked to periodontal disease, such as visfatin, leptin, matrix metalloproteinases (MMP) including MMP-8, MMP-9, and MMP-13, as well as interleukins (IL)-including IL-1β, 1L-2, IL-6, IL-8, and IL-17 (31).

Due to its relative simplicity and lack of invasiveness, GCF has been proposed as a compelling biomarker-based predictive, screening, and diagnostic method (31). The value of biomarkers in the early detection of disease or prediction of at-risk patients has gained interest over traditional diagnostic tools (35, 36). For instance, GCF biomarkers, such as MMPs, IL-1β, and prostaglandin E2 (PGE2) were found to be associated with numerous systemic diseases, including cardiovascular, rheumatological, dermatological, and autoimmune disorders (27, 32). Table 1 summarizes the methods and findings of key studies that investigated the association between GCF biomarkers and the risk of common systemic disorders.

**Table 1:** GCF biomarkers associated with systemic diseases.

Biomarker	Study/Study design/Sample size	Time of Examination	GCF Sampling Method	Biomarker Quantification Method	Findings
MMP-8	Ehlers et al. (37) Case–Control Study (*N* = 40, Cases: 20, Controls: 20)	Within one week of the myocardial infarction	Paper Strips	DentoAnalyzer	GCF MMP-8 concentrations were significantly increased in AMI patients as compared to patients without cardiovascular disease with similar periodontal conditions.
	Valenzuela et al. (38) Exploratory Case–Control Study (*N* = 44, Cases: 23, Controls: 21)	Not Reported	Paper Strips	Multiplex Bead Immunoassay	GCF MMP-8 levels were lower in atopic dermatitis patients as compared to healthy patients.
	Biyikoglu et al. (39) Exploratory Case–Control study (*N* = 40, Cases: 20, Controls: 20)	After a complete periodontal examination	Paper Strips	ELISA	Despite treatment with NSAIDS and corticosteroids, similar GCF MMP-8 levels in patients with rheumatoid arthritis and healthy individuals were found which suggests that rheumatoid arthritis tends to overproduce this enzyme.
MMP-9	Lagana et al. (40) Case–Control Study (*N* = 51, Cases: 28, Controls: 23)	Not Reported	Endodontic Paper Cone	Gelatin Substrate Zymography	Enhanced activity of MMP-13 along with increased levels of active MMP-9 acts as an important biomarker for the early diagnosis of Marfan syndrome.
MMP-13	Lagana et al. (40) Case–Control Study (*N* = 51, Cases: 28, Controls: 23)	Not Reported	Endodontic Paper Cone	Gelatin Substrate Zymography	Enhanced activity of MMP-13 along with increased levels of active MMP-9 acts as an important biomarker for the early diagnosis of Marfan syndrome.
	Biyikoglu et al. (39) Exploratory Case–Control Study (*N* = 40, Cases: 20, Controls: 20)	After a complete periodontal examination	Paper Strips	ELISA	Despite treatment with NSAIDS and corticosteroids, similar GCF MMP-13 levels in patients with rheumatoid arthritis and healthy individuals were found which suggests that rheumatoid arthritis tends to overproduce this enzyme.
IL-18	Valenzuela et al. (41) Cross-Sectional Study (*N* = 81, Cases: 42, Controls: 39)	Not Reported	Paper Strips	Multiplex Bead-Based Immunoassay	GCF IL-18 levels were higher in psoriatic patients as compared to healthy controls.
sNRP-1	Prieto et al. (42) Exploratory Case–Control Study (*N* = 40, Cases: 20, Controls: 20)	After a complete periodontal examination	Paper Strips	ELISA	GCF sNRP-1 concentrations were significantly higher in patients with rheumatoid arthritis as compared to healthy individuals.
E-selectin	Valenzuela et al. (41) Cross-Sectional Study (*N* = 81, Cases: 42, Controls: 39)	Not Reported	Paper Strips	Multiplex Bead-Based Immunoassay	GCF E-selectin levels were lower in psoriatic patients as compared to healthy controls.
SARS-CoV-2 RNA	Gupta et al. (43) Cross-Sectional study (*N* = 33)	After confirmed COVID-19 infection	Microcapillary Pipettes	Real-Time Reverse Transcriptase PCR	SARS-CoV-2 E genes were detected in around 64% of GCF samples, suggesting GCF to be a possible mode of transmission of SARS-CoV-2.
Adrenomedullin	Ertugrul et al. (44) Case–Control Study (*N* = 84, 21 periodontally healthy, 21 with chronic periodontitis, 21 periodontally healthy with DM type 2, and 21 with chronic periodontitis and DM type 2)	After all clinical and radiologic examinations and sampling site selections were performed	Paper Strips	ELISA	GCF adrenomedullin concentrations were significantly increased in individuals with chronic periodontitis and diabetes mellitus.
Resistin	Joshi et al. (45) Comparative Interventional Trial (*N* = 40, Cases: 20, Controls: 20)	Before and 3 months following non-surgical periodontal therapy	Microcapillary Pipettes	ELISA	GCF resistin concentrations were increased in patients with diabetes and periodontitis as compared to individuals with gingivitis.

## Pregnancy and periodontal disease

Periodontal disease is one of the most common chronic inflammatory disorders with a prevalence of 10–60% in adults (46–49). It encompasses both gingivitis and periodontitis. In gingivitis, the inflammatory process is limited to the gingival epithelium while the connective tissue attachment to the teeth remains intact. In periodontitis, the inflammation further affects the supporting connective tissue of the teeth and can progress to cause alveolar bone destruction and teeth loss (50, 51).

The incidence and prevalence of periodontal disease are substantially increased in pregnant women. Around 60–75% of pregnant women may be affected by gingivitis but not necessarily periodontitis as the progression to periodontitis is limited in pregnancy (51). Multiple risk factors have been identified as predictors of risk such as gestational age, maternal age, increased BMI, maternal education and employment status, and smoking (46, 48). Nevertheless, there is no adequate evidence that pregnancy directly causes periodontal disease, rather it appears to increase the severity of a preexisting disease, particularly during the second and third trimesters. Investigated mechanisms include the increased circulatory levels of sex hormones, estrogen and progesterone, and the hormonal modulatory effects of cytokines which can lead to gingival inflammation (51, 52).

In the meantime, periodontal disease has been linked to multiple adverse pregnancy outcomes, including gestational diabetes mellitus, preeclampsia, preterm birth, and low birth weight (34, 46, 48, 50–61). These complications are among the significant causes of perinatal maternal and fetal morbidity and mortality. The etiologic mechanism remains unclear, however, the transfer of periodontal pathogens and inflammatory cytokines from the oral cavity to the uterus and fetoplacental unit is presumed (46). Accordingly, the detection of periodontal disease is therefore imperative for adequate management and patient outcomes.

## The potential of GCF biomarkers in the prediction of pregnancy complications

**Figure 2:**
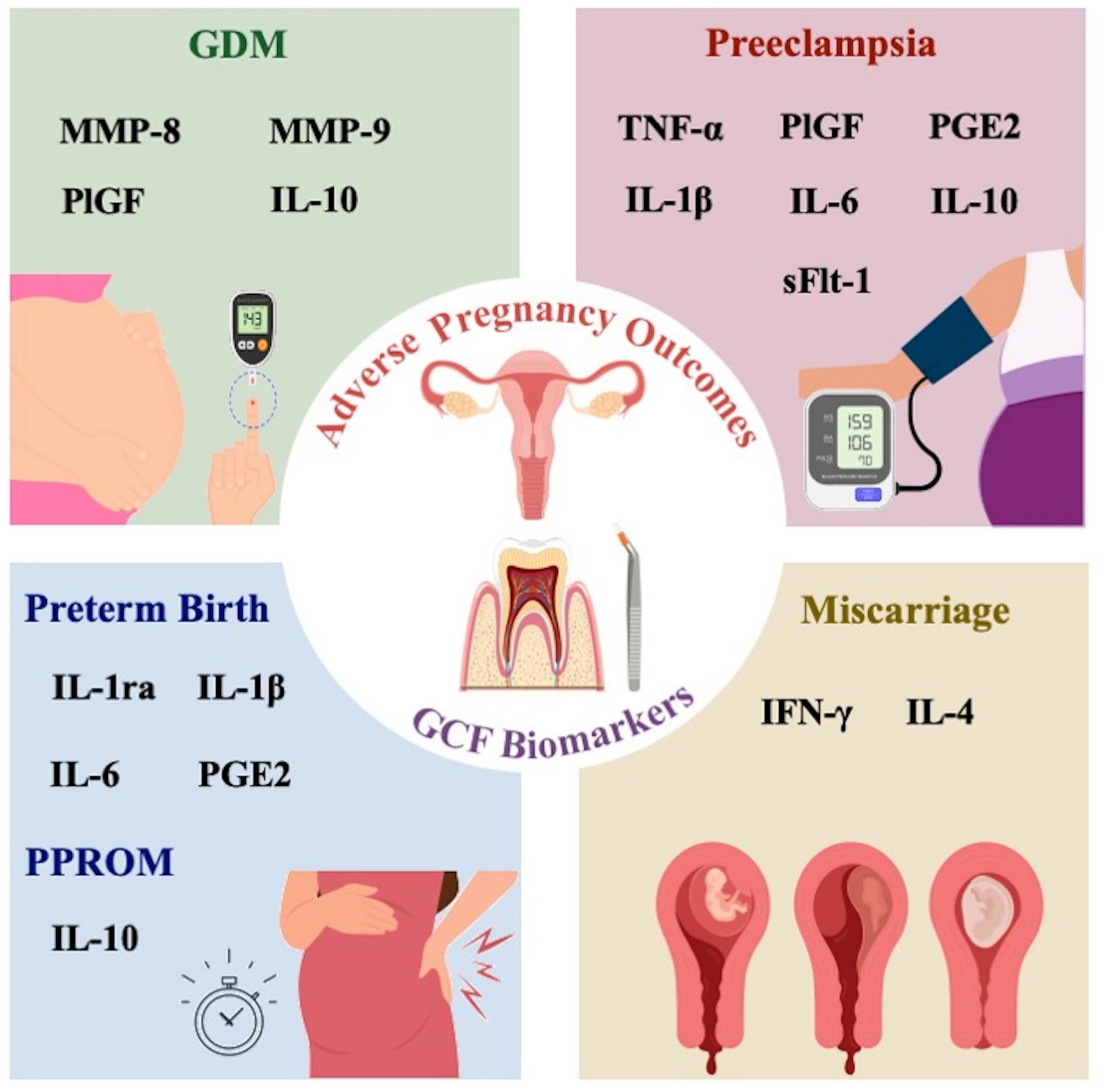
Schematic figure showing different GCF biomarkers that have been reported to be associated with adverse pregnancy outcomes.

Many significant pregnancy adverse outcomes start asymptomatically and are associated with significant morbidity and mortality for both the mother and the fetus. Current conventional screening tests are mostly performed in the second or third trimester when most pregnancy complications are expected to manifest. In recent years, GCF based biomarker testing has been investigated as a potential method that may help in the prediction of pregnancy complications. In the following sections, we review the current evidence that support a potential association of various GCF biomarkers with the risk of common pregnancy specific disorders, as summarized in Table 2 and illustrated in Figure 2.

**Table 2:** GCF biomarkers associated with pregnancy complications.

Biomarker	Study/Study design/Sample size	Gestational age at examination	GCF sampling method	Biomarker quantification method	Findings
MMP-8	Chaparro et al. (62) Prospective Cohort Study (*N* = 358)	11–14 Weeks of Gestation	Papers Strips	Multiplex ELISA Assays	GCF levels of MMP-8 in early pregnancy are elevated in women with severe periodontitis and linked to the development of GDM.
MMP-9	Chaparro et al. (62) Prospective Cohort Study (*N* = 358)	11–14 Weeks of Gestation	Papers Strips	Multiplex ELISA Assays	GCF levels of MMP-9 in early pregnancy are elevated in women with severe periodontitis and linked to the development of GDM.
PGE2	Oettinger-Barak et al. (63) Case Series (*N* = 30, Cases: 15, Controls: 15)	Up to 48 h before delivery	Paper Strips	ELISA	Preeclamptic women showed worse periodontal parameters and significantly increased GCF PGE2 concentrations.
	Perunovic et al. (64) Randomized Cross-Sectional Observational Study (*N* = 120, Cases: 60, Controls: 60)	Within 48 h following delivery; Before completed 37 weeks of gestation for PTB	Paper Strips	ELISA	Women with preterm birth (PTB) showed worse periodontal parameters and substantially elevated GCF concentrations of PGE2 compared with those with full-term birth.
PLAP	Chaparro et al. (65) Prospectively Collected, Retrospectively Stratified Cohort Study (*N* = 412)	11–14 Weeks of Gestation	Paper Strips	ELISA	Increased GCF levels of PLAP were found in patients with preeclampsia (PE).
	Chaparro et al. (66) Case–Control Study (*N* = 30, Cases: 10, Controls: 20)	Up to 24 h once diagnosed with PE; Median of 33 + 1 weeks of gestation for cases; Median of 31 + 3 weeks of gestation for controls	Paper Strips	ELISA	
PlGF	Chaparro et al. (67) Nested Case- Control within a Prospective Cohort Study (*N* = 212)	11–14 Weeks of Gestation	Paper Strips	ELISA	GCF levels of PlGF in the first trimester are substantially elevated in pregnant women with periodontitis who later develop GDM.
IL-1β	Oettinger-Barak et al. (63) Case Series (*N* = 30, Cases: 15, Controls: 15)	Up to 48 h before delivery	Paper Strips	ELISA	Preeclamptic women showed worse periodontal parameters and significantly increased GCF IL-1β concentrations.
	Offenbacher et al. (68) Randomized Control Trial (*N* = 67, Cases: 35, Controls: 32)	Cases: <22 Weeks, Controls: 6 Weeks Postpartum	Paper Strips	ELISA	GCF IL-1β concentrations were increased by around 40% in pregnant women at increased risk of preterm birth.
IL-1ra	Kayar et al. (69) Case–Control Study (*N* = 156, 64 with NB, 45 with PLBW, 47 with IUGR)	Within 24 h of delivery	Paper Strips	ELISA	Women with preterm low birth weight (PLBW) and intrauterine growth restriction (IUGR) showed worse periodontal parameters and lower levels of IL-1ra as compared to women with normal births.
IL-4	Xiao et al. (70) Case–Control Study (*N* = 90, Cases: 50, Controls: 40)	Cases: 5–28 Weeks, Controls: 39–41 Weeks	Paper Strips	ELISA	GCF levels of IL-4 were decreased in patients with recurrent miscarriage and periodontitis as compared to women with uncomplicated pregnancies.
IL-6	Chaparro et al. (71) Case–Control Study (*N* = 54, Cases: 11, Controls: 43)	11–14 weeks of Gestation	Paper Strips	ELISA	Increased GCF concentrations of IL-6 were found in pregnant women with periodontitis who later develop preeclampsia.
	Offenbacher et al. (68) Randomized Control Trial (*N* = 67, Cases: 35, Controls: 32)	Cases: <22 Weeks, Controls: 6 Weeks Postpartum	Paper Strips	ELISA	GCF IL-6 concentrations were increased by two folds in pregnant women at increased risk of preterm birth.
	Perunovic et al. (64) Randomized Cross Sectional Observational Study (*N* = 120 Cases: 60, Controls: 60)	Within 48 h following delivery; Before completed 37 weeks of gestation for PTB	Paper Strips	ELISA	Women with preterm birth (PTB) showed worse periodontal parameters and substantially elevated GCF concentrations of IL-6 compared with those with full-term birth.
IL-10	Ozcaka et al. (72) Cross-Sectional study (*N* = 161, Cases: 96, Controls: 65)	24–28 Weeks of Gestation	Paper Strips	ELISA	GCF IL-10 levels were substantially higher in women with GDM than in non-diabetic pregnant women.
	Stadelmann et al. (73) Prospective Case–Control Study (*N* = 56, Cases: 32, Controls: 24)	Time 1: 20–35 Weeks, Time 2: within 48 h after parturition, Time 3: 4–6 weeks after parturition	Paper Strips	ELISA	GCF concentrations of IL-10 in pregnant women with PPROM were elevated as compared to women with uncomplicated pregnancies.
IFN-γ	Xiao et al. (70) Case–Control Study (*N* = 90, Cases: 50, Controls: 40)	Cases: 5–28 Weeks, Controls: 39–41 Weeks	Paper Strips	ELISA	GCF levels of IFN-γ were elevated in women with recurrent miscarriage and periodontitis as compared to women with uncomplicated pregnancies.
sFlt-1	Chaparro et al. (66) Case–Control Study (*N* = 30, Cases: 10, Controls: 20)	Up to 24 h once diagnosed with PE; Median of 33 + 1 weeks of gestation for cases; Median of 31 + 3 weeks of gestation for controls	Paper Strips	ELISA	Increased GCF levels of sFlt-1 were found in patients with preeclampsia.
TNF-α	Oettinger-Barak et al. (63) Case Series (*N* = 30, Cases: 15, Controls: 15)	Up to 48 h before delivery	Paper Strips	ELISA	Preeclamptic women showed worse periodontal parameters and significantly increased GCF TNF-α concentrations.
Extracellular Vesicles	Monteiro et al. (74) Case–Control Study (*N* = 34, Cases: 11, Controls: 23)	11–14 Weeks of Gestation	Paper Strips	ELISA, Nanoparticle Tracking Analysis, and Transmission Electron Microscopy	GCF levels of extracellular vesicles were elevated in pregnant women who develop GDM later in pregnancy as compared to normoglycemic women.
Glutathione Peroxidase	Canakci et al. (75) Case–Control Study (*N* = 40, Cases: 20, Controls: 20)	Within 48 h before delivery.	Paper Strips	Spectrophotometry	Preeclamptic women with periodontal disease had decreased GCF concentrations of antioxidant enzymes including glutathione peroxidase.
Malondialdehyde					Preeclamptic women with periodontal disease had elevated concentrations of oxidants such as malondialdehyde.
Superoxide Dismutase					Preeclamptic women with periodontal disease had decreased GCF concentrations of antioxidant enzymes including superoxide dismutase.

### Gestational diabetes mellitus

Various studies indicated a potential relationship between periodontal disease and gestational diabetes mellitus (GDM) (34, 50–52, 54, 62, 67, 72, 74, 76–81). Periodontal disease can cause chronic systemic inflammation which is known to increase insulin resistance (76, 81). Moreover, among GCF inflammatory biomarkers, IL-10 was found to be higher in GDM patients compared to non-diabetic pregnant women, and a potential causative relationship was suggested between such inflammatory states and pancreatic beta cell dysfunction (72). Whether this relationship is true or a consequence of gingival inflammation which is likely to occur in GDM patients remains to be investigated.

There is evidence that the development of GDM is associated with increased GCF concentrations of exosomes, which are a group of small extracellular vesicles (EVs) released by the placenta from the 6^th^ week of gestation onwards. The isolation, characterization, and classification of extracellular vesicles obtained from the GCF were first reported by Monterio et al. (74). At 11 to 14 weeks of gestation, greater levels of EVs were found in the GCF of pregnant women who subsequently develop GDM later in pregnancy as compared with normoglycemic pregnant women regardless of periodontal status. This is believed to be due to hyperglycemic and pro-inflammatory conditions in the early pregnancy state of those at-risk women. Accordingly, quantifying the GCF concentrations of EVs in the GCF, early in pregnancy, along with other clinical indicators, may provide a potentially valuable first-trimester screening tool (74).

Placental growth factor (PlGF) is another biomarker released during the vascular development of the placenta. It is a proangiogenic factor that contributes to the growth and maturation of placental vessels. An exploratory nested case–control study demonstrated that the levels of GCF levels of P1GF during the first trimester can be substantially elevated in pregnant women with periodontitis who subsequently develop GDM. Using an algorithm based on the elevated GCF levels of P1GF together with serum glucose levels during first trimester could detect 89.9% of GDM cases, thereby suggesting it as a potential biomarker for GDM screening (67).

Likewise, metalloproteinases (MMPs) are endopeptidases that may play a role in the development of GDM through proteolytic fragmentation of insulin receptors. Accumulating evidence suggests the value of measuring maternal levels of MMP as a potential first trimester screening test for early detection of GDM. A prospective cohort study demonstrated that the levels of GCF MMP-8 and MMP-9 measured between 11 and 14 weeks of gestation were elevated in pregnant women who subsequently develop GDM as compared to uncomplicated pregnancies, stratified by their periodontal inflammatory status and severity of periodontitis (62). Raised levels of these markers in the GCF of GDM patients with periodontal disease were also supported by the findings of Akcali et al. (77).

### Preeclampsia

Preeclampsia remains a major cause of maternal death and severe morbidity worldwide, as such, extensive research efforts are being made toward the early detection of the disease (82). Some of the emerging serum biomarkers in preeclampsia include the soluble fms-like tyrosine kinase-1 (sFlt-1) and PlGF (66, 83). sFlt-1 is a potent antagonist of PlGF that causes vasoconstriction and endothelial damage, which increases the risk of preeclampsia and fetal growth restriction. Low serum concentrations of PlGF and high concentrations of sFlt-1 were reported in patients at high risk of developing preeclampsia later in pregnancy (66, 83). A high serum sFlt-1/PlGF ratio was found to be a more reliable predictor of preeclampsia than either biomarker alone (84). Both biomarkers were detected in the GCF and sFlt-1 concentrations were found to be significantly higher in preeclamptic patients regardless of their periodontal clinical status (66). Based on this evidence, the evaluation of the sFlt-1/PlGF ratio in the GCF in pregnant women can be further studied to establish its potential predictive role for preeclampsia risk.

Placental alkaline phosphatase (PLAP) is another biomarker that has been linked to the risk of preeclampsia. It is a membrane-bound glycoprotein involved in the regulation of placental differentiation, nutrient transport, as well as maternal and fetal metabolism (65). PLAP levels increase as the normal pregnancy progresses, however, in preeclampsia its serum concentrations are much higher most likely due to placental dysfunction (85). Recent studies identified PLAP in the GCF, showing that its concentrations are significantly higher in the GCF of preeclamptic patients in comparison to healthy pregnant women, independent of the periodontal clinical status (65, 66). Therefore, the measurement of GCF levels of PLAP along with blood pressure at 11 to 14 weeks of gestation was suggested as a useful early predictive test for preeclampsia risk (65).

Chronic inflammation is a recognized risk factor for preeclampsia development. Multiple conditions that are linked to chronic inflammation, such as obesity, diabetes mellitus, chronic hypertension, chronic kidney disease, and periodontal disease, have also been associated with an increased risk of developing preeclampsia. Several studies showed an association between periodontal disease and preeclampsia (34, 46, 50–52, 54–58, 63, 66, 71, 85–91). It is believed that periodontal disease may provide a persistent source of inflammatory mediators involved in endothelial dysfunction, a key pathophysiologic finding in preeclampsia. The concentrations of IL-1β, TNF-α, and PGE2 were found to be significantly higher in the GCF of patients with preeclampsia compared to healthy pregnant women (63, 66). Likewise, the elevated levels of IL-6 in the GCF of pregnant patients with periodontitis were associated with a higher risk of subsequent development of preeclampsia (71). Moreover, periodontal disease can reduce the tissue antioxidant capacity and/or increase oxidative stress, another mechanism implicated in the risk of preeclampsia. Low GCF levels of antioxidant enzymes such as superoxide dismutase and glutathione peroxidase, decreased total antioxidant capacity, and increased levels of markers of oxidative stress such as malondialdehyde were detected in preeclamptic patients with periodontal disease (75).

### Preterm birth

Periodontal disease is estimated to be a contributing risk factor for 5–38% of preterm births globally (51). The association between periodontal disease and preterm birth continues to be widely investigated (32, 34, 46–53, 55, 57–60, 68, 89, 90, 92–95). Various studies propose that systemic dissemination of local inflammation due to periodontitis may play a role in the development of preterm birth and ultimately low birth weight (53, 64, 92). PGE2, IL-1β, and IL-6 are inflammatory mediators considered to be the main triggers of labor; they increase gradually throughout pregnancy until reaching a critical concentration for normal labor onset. Local infections, such as periodontal disease, can induce a similar yet premature immunological status due to homeostatic disruption of labor mediators (64).

Several studies assessing the association between periodontal inflammation and preterm birth revealed substantially elevated GCF concentrations of PGE2 in women who delivered prematurely as compared to women who delivered at full term (53, 64, 95–97). Moreover, elevated GCF concentrations of IL-6 were associated with an increased risk of preterm birth (64). A randomized clinical trial demonstrated approximately a 40% increase in GCF levels of IL-1β and a two-fold increase in IL-6 concentrations, which were associated with a significant increase in the risk of preterm delivery (68). Similar findings were reported in another study (98). Meanwhile, decreased GCF levels of other cytokines, such as IL-1 receptor antagonist (IL-1ra), were also linked to preterm birth and poor periodontal status (69).

### Preterm premature rupture of membranes

Preterm premature rupture of the membranes (PPROM) occurs in around 3% of pregnancies and accounts for up to 40% of preterm births. PPROM can be a cause of marked neonatal morbidity and mortality primarily due to prematurity and sepsis (99). Periodontal inflammation is a common finding in patients with premature rupture of the membranes. Elevated GCF concentrations of IL-10 in pregnant women with PPROM were demonstrated as compared to women with uncomplicated pregnancies. Further evidence is required to support the potential role of measuring GCF levels of IL-10 to predict pregnant women at risk of developing PPROM (73).

### Miscarriage

The release of infection-related inflammatory mediators is believed to cause early uterine contractions and thus can increase the risk of miscarriage (51). One case–control study demonstrated higher GCF levels of IFN-γ and lower levels of IL-4 in patients with recurrent miscarriage and periodontitis in comparison to patients with uncomplicated pregnancies. This can possibly cause chronic alteration of the maternal intrauterine immune environment (70). Therefore, the measurement of GCF levels of these two markers together with exploring other candidate biomarkers implicated in the etiology of miscarriage may support the ongoing challenge in the management of patients with recurrent pregnancy loss.

### Small for gestational age

A probable association was suggested between periodontal disease and an elevated relative risk of small for gestational age pregnancies (SGA) explained by the transmission of periodontal bacteria and inflammatory markers to the pregnant uterus. Women delivering infants small for their gestational age exhibited significantly poorer periodontal parameters, however, no correlation was found between concentrations of GCF biomarkers and SGA. Although there is some evidence that the treatment of periodontal disease during pregnancy may reduce the levels of GCF inflammatory cytokines, such as IL-1B, IL-10, IL-12p70, and IL-6; however, there is no adequate evidence that such treatment improves pregnancy outcomes including SGA (48).

### Hyperemesis gravidarum

Hyperemesis gravidarum (HG) is a severe rare pregnancy disorder characterized by persistent nausea and vomiting that is associated with a 5% loss of pre-pregnancy weight and dehydration as often indicated by the detection of ketones in the urine. The etiology of HG remains not well understood but recognized risk factors include multiple or molar pregnancies, hyperthyroidism, young maternal age, very low or high maternal BMI, history of previous HG, and gastroesophageal reflux disease (100, 101). Currently, HG does not have a definitive diagnostic biomarker or screening test. Ketonuria is the most used biomarker in the diagnostic workup for HG, yet its diagnostic accuracy is questionable. Serum biomarkers that have been suggested to have an etiological role in HG include serum levels of hCG, thyroid hormones, leptin, sex hormones, white blood count, and lymphocytes. None of these biomarkers yielded unequivocal results, however, hCG and thyroid hormones may have a potential diagnostic value awaiting further research evidence. Serum *H. pylori* immunoglobulin G demonstrated a significant positive association with HG (102). Similar to other pregnancy disorders discussed above, it could be assumed that the investigation of these potential biomarkers in the GCF may reveal new associations for screening or diagnostic purposes in HG.

### A promising tool awaiting adequate evidence

Presently, GCF-based assays have been applied at the clinical research level. Thus far, there is no routine clinical test available for diagnosis or screening. The research evidence of the reliability and performance of the GCF assays is ongoing. Noteworthy, several studies reviewed in this article have limitations including a small sample size and the lack of solid evidence of a causal relationship between the biomarkers and disease. The current evidence is largely driven by studies investigating the association between GCF-biomarkers and already established complex diseases (63–67, 69, 71, 73–75, 77). Therefore, further well-designed studies are awaited to support further application into the routine clinical setting.

## Conclusion

Introducing novel screening algorithms early in pregnancy that utilize both clinical and biomarker-based assays is of utmost importance to progress the field of high-risk obstetrical care. Growing evidence suggests a promising role of GCF biomarkers in the early detection of several adverse pregnancy outcomes. GCF-based assays have the advantage of being feasible, minimally invasive, and cost-effective. Such feasibility should encourage further focused research with larger prospective studies to confirm the specificity, sensitivity, and predictive value of each candidate GCF biomarker for the pregnancy condition of interest. Multidisciplinary collaboration between obstetricians and periodontists would be pivotal to enable faster progress into routine clinical antenatal care.

## Author contributions

MA and NH equally performed the scientific literature search and wrote the successive drafts of the manuscript. ZB guided the simulated GCF sampling method and reviewed the manuscript. GM edited and critically revised the manuscript. NM conceived the concept, edited, and guided all revisions of the manuscript. All authors reviewed and agreed on the final version of this manuscript.

## Funding

NM is funded by a competitive University of Sharjah grant # 1901090266.

## Conflict of interest

The authors declare that the research was conducted in the absence of any commercial or financial relationships that could be construed as a potential conflict of interest.

## Publisher’s note

All claims expressed in this article are solely those of the authors and do not necessarily represent those of their affiliated organizations, or those of the publisher, the editors and the reviewers. Any product that may be evaluated in this article, or claim that may be made by its manufacturer, is not guaranteed or endorsed by the publisher.
